# Genome-wide transcriptional profiling data from skin of chronic cutaneous lupus erythematosus (CCLE) patients

**DOI:** 10.1016/j.dib.2015.02.024

**Published:** 2015-04-18

**Authors:** R. Dey-Rao, A.A. Sinha

**Affiliations:** Department of Dermatology, University at Buffalo, 6078 Clinical and Translational Research Center, 875 Ellicott Street, Buffalo, NY 14203, United States

**Keywords:** Chronic cutaneous lupus erythematosus, CCLE, Lesional, Non-lesional, Skin, Patients, Gene expression, Transcriptional profiling, Microarray, CEL

## Abstract

Cutaneous features manifest as a wide range of clinically significant, and in many cases disfiguring and debilitating components of lupus erythematosus (LE). While the definitive etiology is in question, multifactorial and polygenic causes are likely to be involved in the production of the characteristic anti-nuclear autoantibody titers and immune cell infiltrates observed in chronic cutaneous LE (CCLE) [1–3]. There is significant overlap of patients with systemic and cutaneous manifestations of LE, which suggests shared pathways and genetic background between the two. We have employed genome-wide microarray technology along with pathway-based analyses to investigate transcriptional differences between lesional and non-lesional skin from CCLE patients to address existing gaps in knowledge regarding disease mechanisms in lupus [4].

## Specifications table

**Table:** 

Subject area	*Dermatology*
Organism/cell	*Homo sapiens*
More specific subject area	*Lesional and non-lesional skin samples from patients with chronic cutaneous lupus erythematosus (CCLE)*
How data was acquired	*Affymetrix GeneChip HG-U95set*
Data format	*Raw data: CEL files*
Experimental factors	*Comparison of lesional and non-lesional skin from CCLE patients*
Experimental features	*We performed both unsupervised and supervised analysis and established differentially expressed genes (DEGs) between lesional and non-lesional skin of CCLE patients. The DEGs were then analyzed by pathway and biological processes-based enrichment analyses via DAVID and Metacore.*
Data source location	*Patients diagnosed with CCLE, and more specifically DLE, were recruited into the study from the Dermatology Outpatient Clinic of New York Presbyterian Hospital, Cornell University.*
Data accessibility	*Data is available with this article*

## Value of the data

• The CEL files included with this document will allow access to genome-wide transcriptional profiling data from lesional and non-lesional skin of CCLE patients, for further analysis.
• The data fill a major gap in knowledge regarding changes that underlie skin specific manifestations in cutaneous lupus.
• The data can be used to link transcriptional profiling data to functional and pathway based analyses to clarify underlying mechanisms of disease.

## 1 Data, materials and methods

The raw data files (CEL files) that were used in the analysis and interpretation in [4] are available in Supplementary information.

Sample IDs are:

LE1001D
LE1001N
LE1003D
LE1003N
LE1006D
LE1008D
LE1009D
LE1009N
LE1010D
LE1010N

Abbreviations:

LE: chronic cutaneous lupus erythematosus
N: non-lesional skin
D: lesional skin

Affymetrix Human genome U95 set GeneChip was used according to manufacturer׳s protocols to analyze gene expression from 6 lesional and 4 non-lesional skin samples of CCLE patients. Expression Console software (Affymetrix) with MAS5 data processing was used to assess the quality of every CEL file in the dataset. Samples determined to be outliers with low quality data and very low percentage present called were removed from the dataset. Default parameters were used to import the gene expression data to Partek Genomics Suite v6.6. Raw data preprocessing was achieved through RMA, including log2 transformation, quantile normalization, background subtraction and median polish probeset summarization to scale mean expression of all 10 arrays. We re-examined QC criteria for our dataset in Partek and found no outliers. Both unbiased and supervised hierarchical cluster analysis were performed and DEGs established. Functional annotation and pathway analysis were performed by standard enrichment analysis via DAVID and Metacore. Further details can be found in the materials and methods and supplementary materials and methods sections [4].

## Footnotes

Appendix A Supplementary data associated with this article can be found in the online version at doi:10.1016/j.dib.2015.02.024.
